# Contribution of Cytology to the Diagnosis of Chediak-Higashi Syndrome

**DOI:** 10.7759/cureus.86597

**Published:** 2025-06-23

**Authors:** Mohammed Ayoub Naamane, Asmaa Harrach, Soukaina Boussif, Hanaa Bencharef, Bouchra Oukkache

**Affiliations:** 1 Hematology Laboratory, Ibn Rochd University Hospital Center, Casablanca, MAR; 2 Faculty of Medicine and Pharmacy, Hassan II University, Casablanca, MAR

**Keywords:** bone marrow cytology, chediak-higashi syndrome, giant azurophilic granules, hematopoietic stem cell transplantation, hemophagocytic lymphohistiocytosis

## Abstract

Chediak-Higashi syndrome (CHS) is a rare autosomal recessive disorder characterized by partial oculocutaneous albinism, recurrent infections, and giant intracytoplasmic granules in leukocytes. Early diagnosis is critical to prevent the onset of severe complications, particularly the accelerated phase. We conducted a descriptive case series in the Hematology Laboratory of Ibn Rochd University Hospital Center, Casablanca. All patients diagnosed with CHS based on cytological analysis were included. Complete blood counts were performed using the SYSMEX® XN-1500 analyzer (Sysmex Corporation, Kobe, Japan), and peripheral blood and bone marrow smears were stained with May-Grünwald Giemsa. Five patients (three girls and two boys) were identified, with a mean age of four years and 11 months. Parental consanguinity was present in all cases. Clinical findings included oculocutaneous albinism (n=4), splenomegaly (n=4), lymphadenopathy (n=3), and recurrent bacterial infections (n=4). Cytological analysis revealed pathognomonic giant granules within granulocytes in all patients. All patients developed hemophagocytic lymphohistiocytosis and succumbed to disease-related complications. In resource-limited settings, cytological evaluation remains a crucial tool for diagnosing CHS when genetic testing is unavailable. Early identification of suggestive clinical features, combined with cytological findings, can facilitate prompt diagnosis and timely initiation of appropriate management, including hematopoietic stem cell transplantation, the only curative treatment available.

## Introduction

Chediak-Higashi syndrome (CHS) is an exceptionally rare autosomal recessive disorder, with an estimated prevalence of fewer than one in a million individuals worldwide. It is characterized clinically by partial oculocutaneous albinism, recurrent pyogenic infections, and neurologic manifestations, while cytologically it presents with pathognomonic giant intracytoplasmic granules in granulocytes and other cells of hematopoietic and non-hematopoietic origin, such as melanocytes, epithelial cells, fibroblasts, neuronal cells, and glandular exocrine cells. The underlying genetic defect involves biallelic mutations in the lysosomal trafficking regulator (LYST) gene, located on chromosome 1q42-43, which plays a crucial role in lysosomal trafficking and exocytosis [1]. Diagnosis typically relies on cytological analysis, particularly peripheral blood and bone marrow smears revealing the characteristic giant granules primarily observed in granulocytes and other hematopoietic cells, such as monocytes and lymphocytes, and may be subsequently confirmed by molecular analysis [1]. In the absence of allogeneic hematopoietic stem cell transplantation (HSCT), CHS often follows a severe course, with most patients developing an accelerated phase similar to hemophagocytic lymphohistiocytosis (HLH), which is often fatal within the first decade of life. In this acute phase, immunosuppressive therapy is used to control the excessive immune activation. This typically includes corticosteroids (such as dexamethasone), etoposide, and sometimes cyclosporine. These agents work together to reduce inflammation, suppress cytokine release, and control hemophagocytic activity. This treatment aims to stabilize the patient and prevent further organ damage, allowing time to proceed to curative HSCT [2]. Only a small number of patients have been reported to survive into adulthood, usually those with the atypical, neurologically predominant variant. While HSCT remains the only curative option for the hematologic manifestations of the disease, it does not reverse existing neurological damage [3]. In resource-limited settings, such as ours, diagnosis depends primarily on cytomorphological evaluation, due to restricted access to genetic testing and specialized immunological assays. To date, published literature on CHS remains scarce, especially in North Africa. Herein, we present the first Moroccan case series of five pediatric patients diagnosed with Chediak-Higashi syndrome based on cytological findings, highlighting the diagnostic value of peripheral blood and bone marrow examination in identifying this life-threatening condition.

## Case presentation

This descriptive case series was conducted in the Hematology Laboratory of Ibn Rochd University Hospital, including all non-consecutive patients diagnosed with Chediak-Higashi syndrome (CHS) between 2020 and 2023. Each patient underwent a comprehensive biological workup comprising a complete blood count (CBC) using the SYSMEX® XN-1500 (Sysmex Corporation, Kobe, Japan) automated analyzer and cytological examination of peripheral blood and bone marrow smears stained with May-Grünwald Giemsa (MGG). We identified five patients, with a sex ratio of two males to three females and a mean age of four years and 11 months (range: 10 months to nine years). Parental consanguinity was present in all cases, and one patient had an affected sibling.

The first patient was a five-year-old female, born to parents with first-degree consanguinity, admitted for febrile splenomegaly and recurrent respiratory infections. Her family history revealed early childhood deaths in a sibling and a cousin, suggestive of a possible inherited immunodeficiency. Clinical examination revealed oculocutaneous albinism with silver-gray hair and hypopigmented skin. Laboratory investigations showed pancytopenia, with normochromic normocytic anemia (hemoglobin 8.4 g/dL), severe neutropenia (320 cells/mm³), and thrombocytopenia (75,000/mm³). Peripheral blood and bone marrow smears revealed heterogeneous giant cytoplasmic granules in granulocytes, which are pathognomonic of CHS. 

The second case was her three-year-old sister, also born to first-degree consanguineous parents, who was evaluated after the index case was diagnosed. She exhibited oculocutaneous albinism with silver-gray hair and hypopigmented skin. Laboratory findings revealed bi-lineage cytopenia, with mild normochromic normocytic anemia (hemoglobin 10.7 g/dL), moderate neutropenia (1110 cells/mm³), and a normal platelet count (331,000/mm³). Cytological analysis of peripheral blood and bone marrow confirmed the presence of heterogeneous giant granules within granulocytic cells. Although she was clinically stable at diagnosis, the biological profile suggested early signs of HLH.

The third case involved a 10-month-old male infant born from a first-degree consanguineous marriage, admitted for recurrent lower respiratory tract infections. The child exhibited failure to thrive with growth and psychomotor delay, oculocutaneous albinism, and a generalized maculopapular rash. On examination, the patient presented with splenomegaly as well as axillary and inguinal lymphadenopathy. Laboratory results showed isolated severe neutropenia (70 cells/mm³), while hemoglobin and platelet levels remained within normal ranges. Cytological examination confirmed the presence of giant granules in granulocytes. The constellation of symptoms and findings was consistent with the diagnosis of CHS associated with HLH.

The fourth case was a 12-month-old female infant, also born from a first-degree consanguineous union, admitted for recurrent infections. Clinical findings included oculocutaneous albinism with hypopigmented skin and silver-gray hair. The biological assessment showed bi-lineage cytopenia, with mild anemia (hemoglobin 11 g/dL), moderate neutropenia (930 cells/mm³), and a normal platelet count (170,000/mm³). Cytological smears demonstrated giant cytoplasmic granules in granulocytic cells. Physical examination revealed splenomegaly and inguinal lymphadenopathy, suggesting a possible diagnosis of hemophagocytic lymphohistiocytosis (HLH). The patient was placed under close clinical monitoring and received supportive treatment.

The fifth and final patient was a nine-year-old male child born of a first-degree consanguineous marriage. He was admitted with persistent fever and generalized lymphadenopathy involving the submandibular, cervical, axillary, and inguinal regions. He also had ocular strabismus and splenomegaly. Laboratory findings revealed pancytopenia, with anemia (hemoglobin 9.5 g/dL), severe neutropenia (370 cells/mm³), and thrombocytopenia (94,000/mm³). Cytological examination of peripheral blood and bone marrow confirmed CHS by the presence of giant granules in granulocytes. He exhibited clinical and biological signs suggestive of HLH and had a prior history of recurrent respiratory infections. A summary of the clinical, hematological, and cytological features of all five cases is presented in Table 1.

**Table 1:** Summary table of clinical and biological data. HLH: hemophagocytic lymphohistiocytosis.

Case	Age/sex	Consanguinity	Clinical features	Complete blood count	Blood and/or bone marrow smear	Complications
1	Five years, female	First-degree consanguinity	Oculocutaneous albinism	Anemia: 8.4 g/dL; Neutropenia: 320/mm³; Thrombocytopenia: 75,000/mm³	Peripheral blood and bone marrow smears: Heterogeneous giant granules in granulocytic cells	HLH: Febrile splenomegaly; recurrent respiratory infections
2	Three years, female	First-degree consanguinity	Oculocutaneous albinism	Anemia: 10.7 g/dL; Neutropenia: 1110/mm³; Platelets: 331,000/mm³	Peripheral blood and bone marrow smears: Heterogeneous giant granules in granulocytic cells	HLH
3	Ten months, male	First-degree consanguinity	Growth and psychomotor delay, Oculocutaneous albinism, Generalized maculopapular rash.	Isolated neutropenia: 70/mm³	Peripheral blood and bone marrow smears: Heterogeneous giant granules in granulocytic cells	HLH: Splenomegaly; inguinal and axillary lymphadenopathy; recurrent respiratory infections
4	Twelve months, female	First-degree consanguinity	Oculocutaneous albinism	Anemia: 11 g/dL; Neutropenia: 930/mm³; Normal platelets: 170,000/mm³	Peripheral blood and bone marrow smears: Heterogeneous giant granules in granulocytic cells	HLH: Splenomegaly; inguinal lymphadenopathy
5	Nine years, male	First-degree consanguinity	Ocular strabismus	Anemia: 9.5 g/dL; Neutropenia: 370/mm³; Thrombocytopenia: 94,000/mm³	Peripheral blood and bone marrow smears: Heterogeneous giant granules in granulocytic cells	HLH: Fever; submandibular, cervical, axillary and inguinal lymphadenopathy; splenomegaly; recurrent respiratory infections

**Figure 1:**
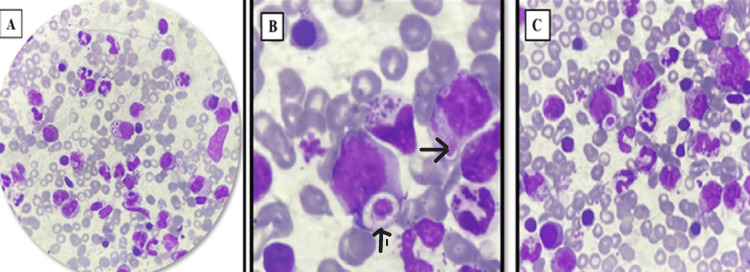
Bone marrow smears (A)-(C) (magnification ×100) showing large abnormal intracytoplasmic inclusions suggestive of Chediak-Higashi syndrome in granulocytic cells.

Cytomorphological analysis revealed the presence of giant granules in leukocytes in all five patients (Figure 1). All patients developed hemophagocytic lymphohistiocytosis (HLH), and four experienced recurrent bacterial infections. Unfortunately, all patients succumbed to complications of the disease.

## Discussion

The Chediak-Higashi Syndrome (CHS) was first described in 1943 by Cuban pediatrician Beguez Cesar, who noted neutropenia and abnormal cytoplasmic granules in leukocytes. A more comprehensive characterization followed in 1948 with Steinbrinck’s description of a second case. In 1952, Chediak highlighted the hematological features of the disease, and in 1953, Higashi described the presence of large peroxidase-containing granules in patient cells [1].

CHS is a rare autosomal recessive disorder caused by mutations in the LYST gene (also known as CHS1) located on chromosome 1q42-43 [4]. Fewer than 500 cases have been reported in the literature over the past 15 to 20 years [5,6].

The LYST gene mutation disrupts intracellular trafficking, leading to the accumulation of giant lysosomal granules and impaired exocytosis in immune cells and melanocytes. This defect results in partial oculocutaneous albinism and impaired cytotoxic function of both natural killer (NK) cells and cytotoxic T lymphocytes. Microscopically, pathognomonic giant granules are observed in several cell types, including neutrophils and melanocytes [6].

Two distinct clinical phenotypes are recognized: a classic, severe childhood-onset form characterized by recurrent infections and an "accelerated phase." A milder, adult-onset form marked by progressive neurologic deterioration [7].

Clinically, CHS typically presents with partial oculocutaneous albinism, silvery or blond hair, photophobia, nystagmus, recurrent pyogenic infections affecting the skin and respiratory tract, bleeding diathesis, and, in some cases, peripheral neuropathy, developmental delay, leukemia, or neoplasia. Without intervention, mortality is common before the age of seven [8].

The observed albinism is secondary to hypopigmentation resulting from the presence of abnormally large melanosomes within melanocytes, which hinders uniform melanin distribution across hair, skin, iris, and the fundus [9]. Hyperpigmentation in CHS is uncommon but has been documented. A lack of awareness of this atypical finding may lead to diagnostic delay by suggesting alternative photosensitivity syndromes [10].

The increased susceptibility to infections is attributed to defective cytotoxicity of NK and CD8+ T cells, as well as impaired chemotaxis and bactericidal function of granulocytes. Antibody production remains intact, and infections typically respond to antibiotics, albeit slowly. The most common pathogens include *Staphylococcus aureus* and β-hemolytic Streptococcus, with gram-negative bacilli, Candida, and Aspergillus playing a more limited role [11].

Hemorrhagic manifestations in CHS are primarily due to functional platelet defects, specifically a deficiency in dense granules, though thrombocytopenia may be evident during the accelerated phase [11].

Between 50% and 85% of patients develop an accelerated phase, a life-threatening lymphohistiocytic infiltration characterized by fever, jaundice, hepatosplenomegaly, lymphadenopathy, pancytopenia, coagulopathy, and multi-organ involvement consistent with hemophagocytic lymphohistiocytosis (HLH) [12]. The precise pathophysiological mechanism underlying HLH in CHS remains unclear, though CTLA4, an immune checkpoint regulator, has been proposed as a possible contributor [4].

Neurological manifestations were initially emphasized by Myers et al. [13] and further detailed by Blume and Wolff [11], with neuropathologic changes extensively described by Sung et al [14]. These include a wide range of signs, such as peripheral and cranial neuropathies, autonomic dysfunction, seizures, sensorimotor deficits, diminished reflexes, unsteady gait, abnormal EEG/EMG findings, and reduced nerve conduction velocities. In early stages, neurodevelopmental delays, learning disabilities, and attention-deficit symptoms are nearly universal, indicating impaired neurodevelopment. A second neurological phase typically emerges in late adolescence and is characterized by progressive deterioration.

In our series, diagnosis was confirmed at a mean age of four years and 11 months, comparable to the mean age of six years reported in the literature for patients not undergoing hematopoietic stem cell transplantation (HSCT). A Moroccan study by Boudarbala et al. reported a similar mean age of four years and two months [15], while Benchidmi et al. documented a single case at age 12 [16].

Consanguinity was present in all cases, in line with the literature, where familial consanguinity is reported in up to 50% of cases. Complete oculocutaneous albinism was observed in four patients. In a cohort of 54 cases, Blume and Wolff reported 38 cases with complete albinism, seven with partial involvement, and two without pigmentary changes [11]. Boudarbala et al. noted partial albinism in two of three reported cases, with one lacking pigmentary sign [15,16].

Approximately 85% of patients progress to an accelerated phase, presenting with systemic lymphohistiocytic infiltration, fever, jaundice, hepatosplenomegaly, lymphadenopathy, cytopenias, hemorrhagic complications, and neurologic symptoms, consistent with findings from our study [15,16].

The diagnosis is established by identifying pathogenic variants in the LYST gene alongside compatible clinical features. Conventional genetic testing by Sanger sequencing identifies most mutations, though certain variants may remain undetected [17]. Typically, patients with the classic CHS phenotype harbor two severe LYST mutations, whereas milder cases often have one severe and one hypomorphic (e.g., missense) variant. While LYST expression in fibroblasts may predict non-neurological disease severity, neurologic complications appear to be genotype-independent.

Given the limited access to genetic testing in our setting and the high cost of enzymatic assays, bone marrow cytology remains a pivotal diagnostic tool. This is consistent with findings from other Moroccan series, including those by Boudarbala et al. [15] and Benchidmi et al [16].

Peripheral blood analysis may reveal anemia, thrombocytopenia, leukocytosis, or leukopenia. Confirmation relies on identifying pathognomonic giant azurophilic granules within granulocytes. These may be missed by automated hematology analyzers. Bone marrow evaluation may also show similar inclusions in myeloid precursors. Bone marrow evaluation may also reveal similar inclusions within myeloid precursors, resembling pseudo-Chediak-Higashi granules [3].

Management of CHS is largely supportive, involving the use of broad-spectrum antibiotics for infections and blood product transfusions to address cytopenias and hemorrhagic risks. Once the accelerated phase sets in, the prognosis is poor despite chemotherapy (e.g., etoposide, corticosteroids, intrathecal methotrexate), with transient responses and frequent relapses. Historically, mortality was inevitable due to infectious or bleeding complications.

Over the past 15 years, allogeneic hematopoietic stem cell transplantation (HSCT) has emerged as the definitive treatment, capable of correcting hematologic and immunologic abnormalities. However, it does not prevent the progression of neurologic dysfunction, which remains a major long-term morbidity [18].

## Conclusions

This first Moroccan report of five cases of Chediak-Higashi syndrome highlights the diagnostic utility of cytology, particularly through the identification of pathognomonic giant granules, in settings with limited access to LYST gene analysis. Early biological evaluation in patients presenting with cytopenia and suggestive clinical signs is essential to enable timely diagnosis and management, ideally prior to progression to the accelerated phase. Immunosuppressive therapy, including corticosteroids, etoposide, and, in some cases, cyclosporine, plays a key role in controlling this critical phase. Allogeneic hematopoietic stem cell transplantation remains the only curative treatment to prevent disease advancement and improve long-term outcomes.
